# Mechanism of biotin carboxylase inhibition by ethyl 4-[[2-chloro-5-(phenylcarbamoyl)phenyl]sulphonylamino]benzoate

**DOI:** 10.1080/14756366.2021.1994558

**Published:** 2021-12-11

**Authors:** Matthew K. Craft, Grover L. Waldrop

**Affiliations:** Department of Biological Sciences, Louisiana State University, Baton Rouge, LA, USA

**Keywords:** Antibiotics, acetyl-CoA carboxylase, fatty acid synthesis, molecular docking

## Abstract

The rise of antibacterial-resistant bacteria is a major problem in the United States of America and around the world. Millions of patients are infected with antimicrobial resistant bacteria each year. Novel antibacterial agents are needed to combat the growing and present crisis. Acetyl-CoA carboxylase (ACC), the multi-subunit complex which catalyses the first committed step in fatty acid synthesis, is a validated target for antibacterial agents. However, there are at present, no commercially available antibiotics that target ACC. Ethyl 4-[[2-chloro-5-(phenylcarbamoyl)phenyl]sulfonylamino]benzoate (SABA1) is a compound that has been shown to have antibacterial properties against *Pseudomonas aeruginosa* and *Escherichia coli*. SABA1 inhibits biotin carboxylase (BC), the enzyme that catalyses the first half reaction of ACC. SABA1 inhibits BC via an atypical mechanism. It binds in the biotin binding site in the presence of ADP. SABA1 represents a potentially new class of antibiotics that can be used to combat the antibacterial resistance crisis.

## Introduction

In a 2020 report, the World Health Organisation called antimicrobial resistance (AMR) “a major threat to human health with significant global economic and security implications”^1^. In the United States, an estimated 2.8 million patients contract AMR infections each year. Of those 2.8 million infections, over 35 000 cases result in death^2^. The economic loss from AMR is difficult to quantify, but estimates are as high as $55 billion (2008 dollars) per year in the U.S. alone^3^. The problem of AMR is not limited to the United States. In 2015, there were an estimated 670 000 AMR infections leading to approximately 33 000 deaths in the European Union (EU) and European Economic Area^4^. The ESKAPE pathogens (*Enterococcus faecium, Staphylococcus aureus, Klebsiella pneumoniae, Acinetobacter baumannii, Pseudomonas aeruginosa*, and *Enterobacter* spp) are particularly problematic. These six pathogens are the leading cause of nosocomial infections worldwide and are known for their ability to “escape” destruction by many current antibiotics^5–8^. Of those six bacteria, four of them (*Klebsiella pneumoniae, Acinetobacter baumannii, Pseudomonas aeruginosa*, and *Enterobacter)* are Gram-negative. Gram-negative bacteria are generally more resistant to antibiotics than Gram-positive organisms, largely because of their outer membrane^9–11^. In order to combat the present and growing threat of AMR, new classes of antibiotics need to be developed^12–14^. While a few novel classes have been developed this past decade, they are not enough to meet the current demand. More antibiotics still need to be developed^15^.

The fatty acid synthesis type-II (FASII) pathway is a validated target for antibiotic development against Gram-negative bacteria^16–18^. In Gram-negative organisms FASII, in addition to its normal role of synthesising saturated fatty acids, is required for the production of β-hydroxy-fatty acids, which are necessary for the synthesis of the lipid A component of lipopolysaccharides (LPS). LPS is a major constituent of the outer membrane of Gram-negative bacteria^19–21^.

**Scheme 1.**
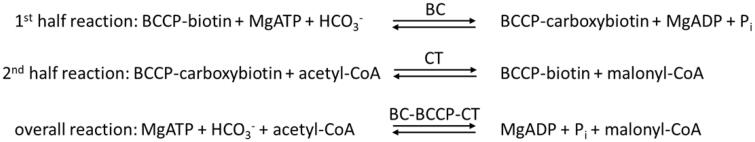
Reactions catalysed by ACC.

Acetyl-CoA carboxylase (ACC) is the multi-subunit complex that catalyses the first committed step in fatty acid synthesis^22^. The complex catalyses the two half reactions, shown in Scheme 1. In the first half reaction, biotin carboxylase (BC) catalyses the ATP dependent carboxylation of the vitamin biotin, which is covalently attached to biotin carboxyl carrier protein (BCCP). This forms a carboxybiotin intermediate. In the second half reaction, carboxyltransferase (CT) transfers the carboxyl group from the carboxybiotin intermediate onto acetyl-CoA, forming malonyl-CoA. The BC and CT subunits retain their activity when isolated separately and can utilise free biotin as a substrate^22^^,^^23^.

**Figure 1.**
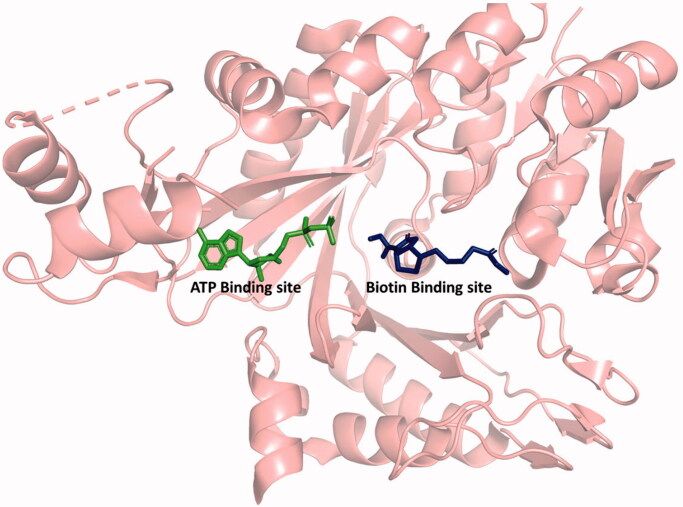
Structure of BC showing the ATP and biotin binding sites. ATP, ADP, aminooxazole, benzimidazole carboxamide, and pyridopyrimidine bind in the ATP binding site. Biotin, bicarbonate, and phosphate bind in the biotin binding site. Generated with PyMOL.

Both CT and BC are the targets of antibacterial compounds. The natural products andrimid and moiramide B both target CT and are competitive versus malonyl-CoA^24^. There are three classes of compounds that inhibit BC: the aminooxazoles^25^, the benzimidazole carboxamides^26^, and the pyridopyrimidines^27^. All three are synthetic compounds that bind in the ATP-binding site and are competitive versus ATP (Figure 1).

**Figure 2.**
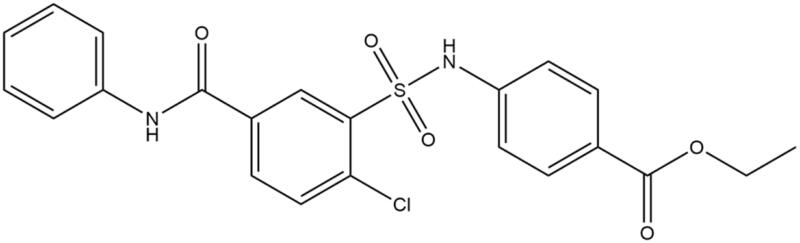
Structure of SABA1.

In 2015 a group from Microbiotix, Inc. developed an *in vivo* high-throughput screen capable of identifying FASII inhibitors in *Pseudomonas aeruginosa* and *Escherichia* coli^28^. That screen identified five compounds that selectively inhibit FASII^28^. Two of those compounds share a sulfonamidobenzamide (SABA) core structure^28^. The more potent of those two compounds is ethyl 4-[[2-chloro-5-(phenylcarbamoyl)phenyl]sulfonylamino]benzoate (SABA1) (Figure 2). SABA1 has a MIC of 0.45–0.9 µM against efflux compromised *E. coli* (*ΔtolC::tet*) and prolongs the doubling time of the efflux compromised *P. aeruginosa* strain PAO397 by 30%^28^. They determined that the antibacterial action of SABA1 is due to inhibition of ACC. It has an IC_50_ of 4.0 µM against *E. coli* ACC^28^ and mapping of resistant mutations suggests that SABA1 targets BC^28^.

In this report, we definitively establish that SABA1 inhibits BC. Instead of binding in the ATP-binding site like all other known inhibitors of BC with antibacterial properties, SABA1 binds in the biotin binding site. We further demonstrate that SABA1 inhibits BC via an atypical mechanism where SABA1 binding is strongly enhanced by the presence of ADP.

## Materials and methods

All reagents for protein expression, purification, and kinetic analysis were purchased from MilliporeSigma, Fischer Scientific, Bio-Rad, New England BioLabs, or Thermo Scientific except where noted. SABA1 was purchased from Hit2Lead.

### Protein expression

Biotin carboxylase with an N-terminal His tag^29^, biotin carboxylase carrier protein with an N-terminal His tag^30^, and carboxyltransferase with a His tag on the N-terminus of the α-subunit^31^ were expressed separately as previously described, with the following modifications. Cells from glycerol stocks were streaked for isolation on a Luria broth (LB) plate with appropriate antibiotics. A 50 mL culture of LB with an appropriate antibiotic was inoculated with an isolated colony and incubated overnight with shaking at 37 °C. The overnight culture was used to inoculate 0.5 L of LB medium in a 2.0-L flask. The cells were grown at 37 °C, shaking at 200 rpm until mid-log phase. Cells were induced with 50 µM Isopropyl β-d-1-thiogalactopyranoside (IPTG). Shaking was decreased to 150 RPM, and the incubation was continued for 18 h at room temperature. Cells were harvested by centrifugation and resuspended in a minimal amount of Buffer A (5 mM imidazole, 25 mM NaPO_4_, 500 mM NaCl, pH 8.0). The resuspended cell pellet was then either frozen or purified immediately.

### Protein purification

Cells were lysed by sonication in the presence of DNase. The resultant lysate was clarified by centrifugation at 4 °C. The clarified lysate was loaded onto a Ni-NTA column pre-equilibrated with Buffer A. The column was washed with 200 mL of Buffer A then 200 mL of NPI-45 (45 mM imidazole, 25 mM NaPO_4_, 500 mM NaCl, pH 8.0). Protein was eluted with NPI-500 (500 mM imidazole, 25 mM NaPO_4_, 500 mM NaCl, pH 8.0) and collected in 1 mL fractions. Purity was determined by SDS-PAGE and pure fractions were pooled. The pooled fractions were first dialysed against 10 mM Hepes pH 8.0, 150 mM KCl, 1 mM EDTA, to remove any residual nickel, then 10 mM Hepes pH 8.0, 150 mM KCl. The pure protein was concentrated, aliquoted, and stored at −80 °C.

### Kinetic assays

For inhibition studies that varied ATP or biotin, initial velocities were measured as previously reported^30^. In brief, pyruvate kinase and lactic dehydrogenase were used to couple ADP production to NADH oxidation, which can be measured spectrophotometrically at 340 nm. For inhibition studies varying ADP, activity was determined by following the production of inorganic phosphate (P_i_) using the EnzChek^®^ Phosphate Assay Kit (Molecular Probes) according to the manufacturer’s protocol. In brief, purine nucleoside phosphorylase converts 2-amino-6-mercapto-7-methylpurine riboside to ribose 1-phosphate and 2-amino-6-mercapto-7-methylpurine in the presence of P_i_. This reaction is monitored at 360 nM^32^. Kinetic data were collected on a Cary60 UV-Vis spectrophotometer (Agilent Technologies)

#### Biotin carboxylase inhibition assays

To determine the inhibition pattern versus ATP, ATP was varied from 0.05 mM to 1.0 mM. SABA1 was held at fixed concentrations of 0.0 µM, 75 µM, or 150 µM. Biotin was held constant at 40 mM. To determine the inhibition pattern versus biotin, biotin was varied from 20 mM and 200 mM. SABA1 was held at fixed concentrations of 0.0 µM, 75 µM, or 150 µM. ATP was held constant at 0.2 mM. Each reaction was started with the addition of enzyme.

To determine the inhibition pattern versus ADP using the P_i_ assay, ADP was varied from 0.19 µM to 19 µM. ATP was held constant at 0.6 mM, biotin was held constant 40 mM, and SABA1 was held constant at 100 µM. Each reaction cocktail was incubated in the presence or absence of SABA1 for 10 min. The reaction was started by addition of biotin.

#### Multiple inhibition assays

To determine the multiple inhibition pattern with respect to ATP, aminooxazole was varied from 0.2 µM to 3.2 µM. SABA1 was held at fixed concentrations of 0.0 µM, 75 µM, or 150 µM. ATP and biotin were held constant at 0.2 mM and 40 mM, respectively. To determine the multiple inhibition pattern with respect to biotin, desthiobiotin was varied from 1.0 mM to 20 mM. SABA1 was held at fixed concentrations of 0.0 µM, 75 µM, or 150 µM. ATP and biotin were held constant at 0.2 mM and 40 mM, respectively.

#### ACC assays

As stated above, the components of ACC were purified separately. Purified BC, BCCP, and CT were combined in a 1:2:1 molar ratio and allowed to equilibrate for 1 h prior to assaying ACC. To determine the inhibition pattern versus ATP, ATP was varied from 1.0 µM to 16 µM. SABA1 was held at fixed concentrations of 0.0 µM, 50 µM, or 100 µM. Acetyl-CoA was held constant at 0.20 mM. The reaction was started by the addition of enzyme.

### Kinetic analysis

Data for inhibition studies varying ATP or biotin were analysed by nonlinear regression using the programs of Cleland^33^. Competitive inhibition data were fitted to Equation (1) where *v* is the experimentally determined velocity, V is maximal velocity, A is substrate concentration, K_m_ is the Michaelis constant, I is inhibitor concentration and K_is_ is the inhibition constant for the slope.

(1) $$v=\frac{\mathrm{VA}}{K_{m}(1+\frac{I}{K_{\mathrm{is}}})+A}$$

Non-competitive inhibition data were fitted to Equation (2) where *v* is the experimentally determined velocity, V is maximal velocity, A is substrate concentration, K_m_ is the Michaelis constant, I is inhibitor concentration and K_ii_ is the inhibition constant for the intercept and K_is_ is the inhibition constant for the slope.

(2) $$v=\frac{\mathrm{VA}}{K_{m}(1+\frac{I}{K_{\mathrm{is}}})+A(1+\frac{I}{K_{\mathrm{ii}}})}$$

Multiple inhibition data were fitted to Equation (3) where *v* is the experimentally determined velocity, V is maximal velocity, L and J are the concentration of the two inhibitors, K_L_ and K_J_ are the apparent inhibition constants of those respective inhibitors, and α is the interaction factor of the two inhibitors.

(3) $$v=\frac{V}{1+\frac{L}{K_{L}}+\frac{J}{K_{J}}+\frac{\mathrm{LJ}}{\alpha K_{L}K_{J}}}$$

Data for inhibition studies varying ADP were analysed by nonlinear regression using the program Enzfitter^34^. Apparent inhibition constants were obtained using Equation (4) where *v* is the velocity in the presence of inhibitor, *v_o_* is the uninhibited velocity, K_i_′ is the apparent inhibition constant, and I is the concentration of inhibitor.

(4) $$v=\frac{v_{o}}{1+\frac{I}{K_{i}`}}$$

The apparent inhibition constants were fitted then to Equation (5). K_i_′ is the apparent inhibition constant, K_ii_ is the real inhibition constant for the intercept, S is the substrate concentration, and K_m_ is the Michaelis constant.

(5) $$K_{i}^{'}=\frac{K_{\mathrm{ii}}(S+K_{m})}{S}$$

### Docking simulations

The structure of SABA1 was obtained from the ZINC database^35^ in SDF format. A single subunit of BC (PDB ID: 4RZQ or 4MV3) was used as the protein model for docking. Both the protein receptor and ligand were prepared for docking using AutoDockTools^36^. The box size for ligand docking, 13.337 Å, was determined by eBoxSize^37^. The box was centred on the position of the N1 nitrogen of biotin. The carboxybiotin analogue, N1′-methoxycarbonyl biotin methyl ester, which binds in the biotin binding site^38^, was docked as a reference compound. Docking was performed using AutoDock Vina^39^. Open babel^40^ was used to convert the ligand from PDBQT format to PDB format for viewing. PyMOL^41^ was used to visualise binding interactions.

## Results

### Inhibition patterns

**Figure 3.**
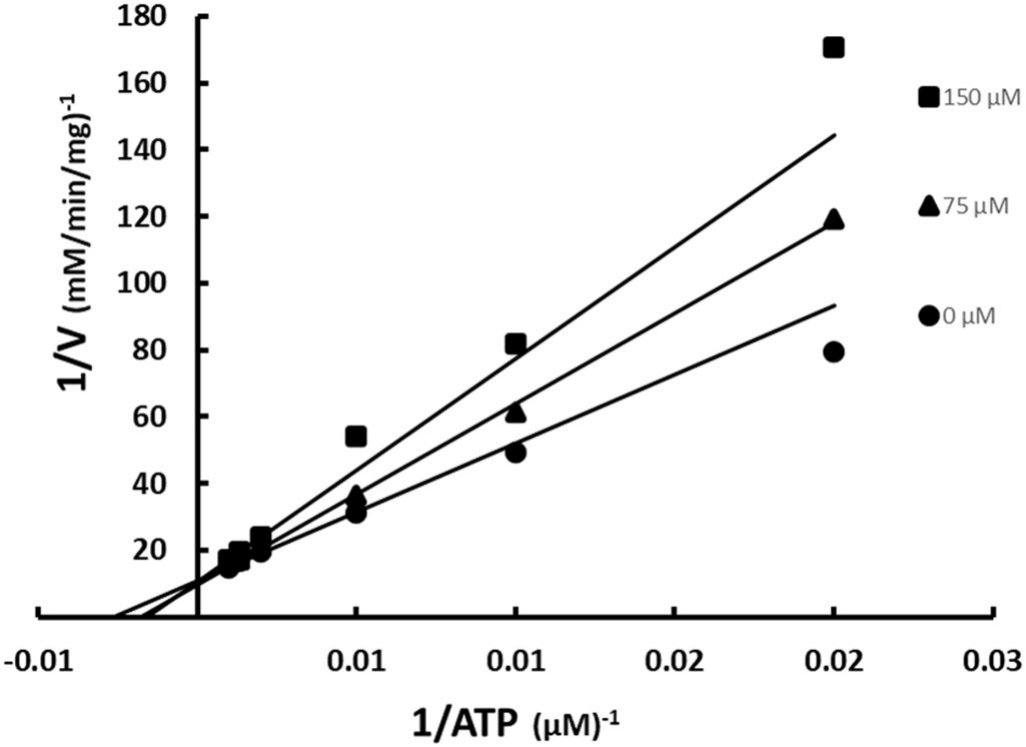
Inhibition of BC by SABA1 with respect to ATP. The concentration of ATP was varied at fixed increasing concentrations of SABA1. Biotin was held constant at subsaturating levels. Curves are the best fit of the data to Equation (1). Points are the experimentally obtained velocities.

**Figure 4.**
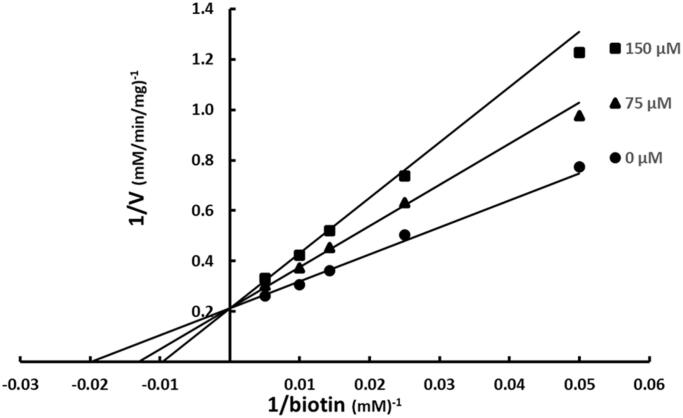
Inhibition of BC by SABA1 with respect to biotin. The concentration of biotin was varied at fixed increasing concentrations of SABA1. ATP was held constant at subsaturating levels. Curves are the best fit of the data to Equation (1). Points are the experimentally obtained velocities.

ATP was varied at fixed increasing concentrations of SABA1 while holding the other substrate biotin constant at subsaturating levels. SABA1 showed a competitive inhibition pattern versus ATP with a K_is_ of 242 ± 32 µM (Figure 3). When biotin was varied at fixed increasing concentrations of SABA1 and ATP was held constant at subsaturating levels a competitive inhibition pattern with a K_is_ of 142.0 ± 12.3 µM was also observed (Figure 4).

### Multiple inhibition analysis

Competitive inhibition means that binding of the inhibitor and the substrate to the enzyme are mutually exclusive. This usually indicates that the inhibitor and substrate bind in the same location. The simple explanation for the competitive inhibition patterns observed for SABA1 suggest the inhibitor can bind in either the ATP or biotin binding sites, or both sites simultaneously. To determine if SABA1 can bind in the ATP binding site, biotin binding site or both, multiple inhibition analysis was performed as described by Yonetani and Theorell^42^. Multiple inhibition analysis is used to define the topological relationship between two different enzyme inhibitors^42^. Initial velocities are measured while one inhibitor is varied against fixed increasing concentrations of the second inhibitor. The substrates are held constant at subsaturating levels.

**Figure 5.**
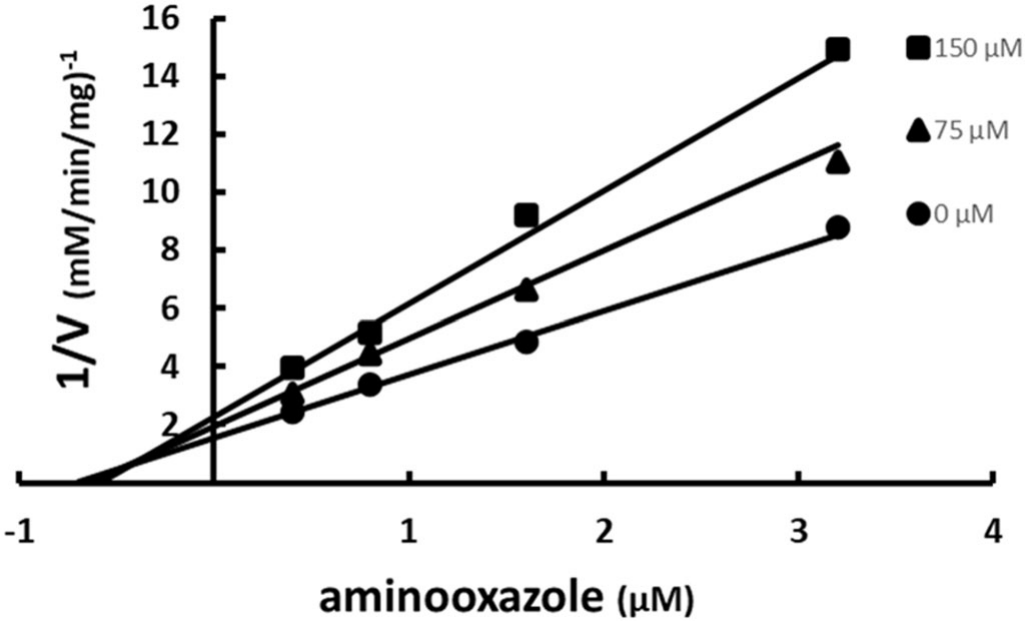
Multiple inhibition pattern for aminooxazole and SABA1. The concentration of aminooxazole was varied at fixed increasing concentrations of SABA1. ATP and biotin were held constant at subsaturating levels. Curves are the best fit of the data to Equation (3). Points are the experimentally obtained velocities.

The possibility of SABA1 binding in the ATP binding site was examined with the BC inhibitor aminooxazole. Aminooxazole has been shown crystallographically to bind in the ATP site^25^. When aminooxazole was varied at fixed increasing concentrations of SABA1, an intersecting pattern was observed (Figure 5). An intersecting pattern indicates that both inhibitors, aminooxazole and SABA1, bind to the enzyme simultaneously. This means that the SABA1 and aminooxazole/ATP binding sites are topologically distinct and therefore SABA1 does not bind in the ATP site. When the data were fitted to Equation (3) an α value of 0.6 was calculated. An intersecting pattern with an α value greater than zero but less than one means the two inhibitors bind synergistically^42^.

**Figure 6.**
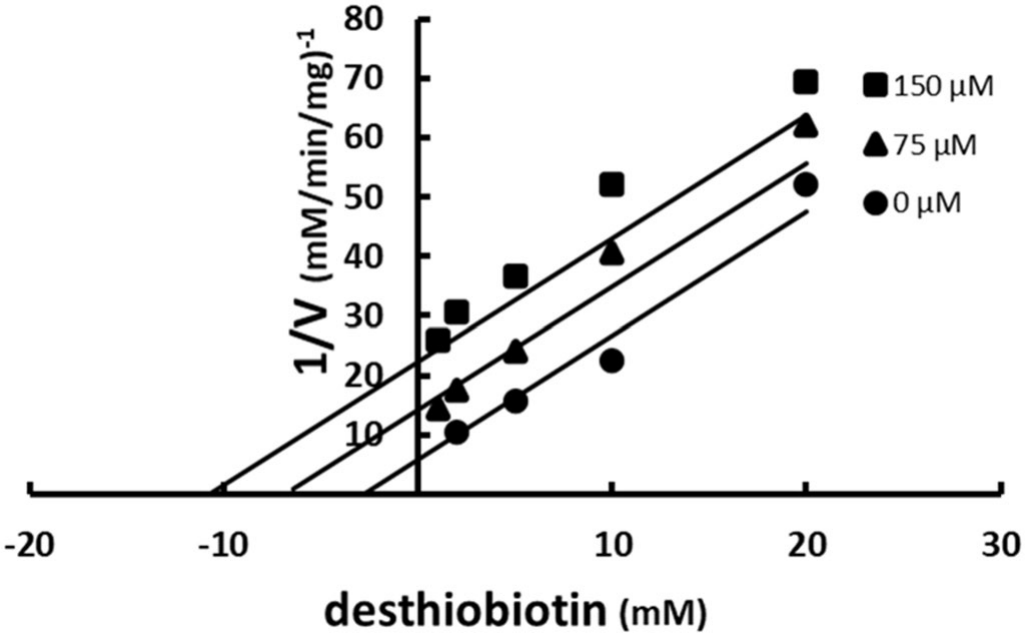
Multiple inhibition pattern for desthiobiotin and SABA1. The concentration of desthiobiotin was varied at fixed increasing concentrations of SABA1. ATP and biotin were held constant at subsaturating levels. Curves are the best fit of the data to Equation (3). Points are the experimentally obtained velocities.

Binding of SABA1 to the biotin-binding site was assessed with desthiobiotin. Desthiobiotin is a slow alternate substrate of BC (0.2% activity of biotin)^43^. A slow alternate substrate can be treated as a dead-end inhibitor if its activity is less than or equal to 2.0% of the true substrate^44^. When desthiobiotin was varied at fixed increasing concentrations of SABA1, a parallel pattern was observed (Figure 6). This indicates that SABA1 and desthiobiotin bind in a mutually exclusive manor. In other words, SABA1 binds in the biotin binding site.

### Inhibition versus ADP

**Scheme 2.**
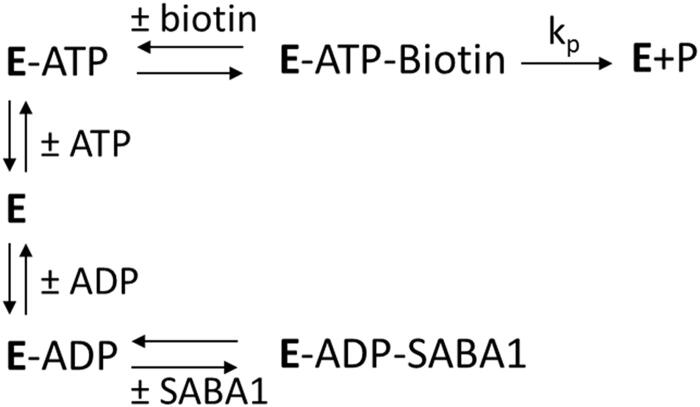
Reaction scheme showing SABA1 binding to the ADP-bound enzyme.

**Figure 7.**
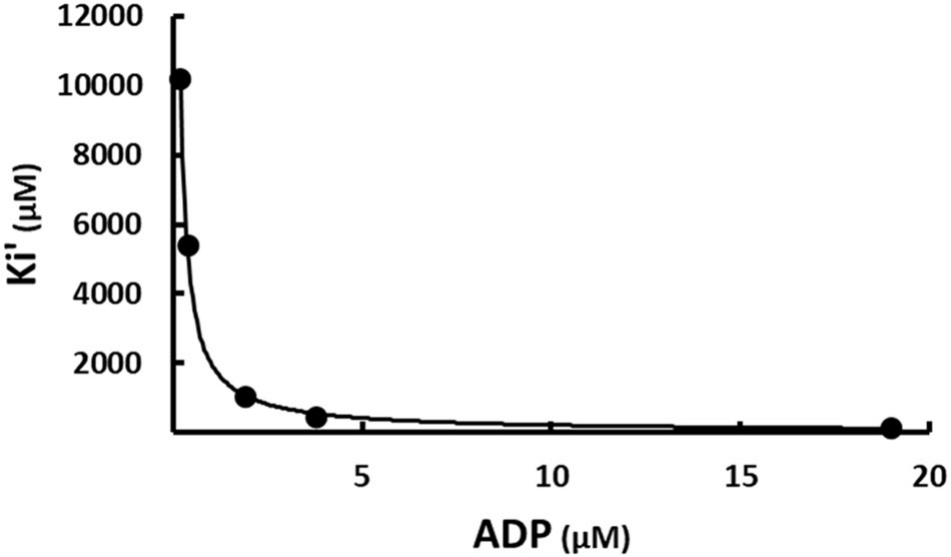
The effect of ADP on the apparent inhibition constant of SABA1. The curve is the best fit of the data to Equation (5), indicating uncompetitive inhibition. Points are the experimentally obtained velocities.

The apparent inconsistency of SABA1 binding in the biotin site, but also exhibiting competitive inhibition versus ATP suggests an inhibition mechanism more intricate than simple competition between SABA1 and biotin. An alternative mechanism for SABA1 inhibition starts by noting that the kinetic mechanism of biotin carboxylase is ordered, with ATP binding before biotin^45^ and that free BC can bind ADP^38^. SABA1 could inhibit BC by binding to the ADP bound form of the enzyme, blocking the addition of biotin (Scheme 2). This is the same mechanism by which the antibacterial agent triclosan inhibits enoyl (acyl carrier protein) reductase (EACPR) which catalyses the reduction of the double bond intermediate in fatty acid synthesis^46^. Like BC, EACPR has an ordered mechanism of substrate binding, with NADH binding before the enoyl substrate^47^. Triclosan binds following the addition of NAD^+ ^^48^ and binds in the enoyl substrate site^49^. The inhibitor exhibits competitive inhibition versus NADH but uncompetitive inhibition versus NAD^+ ^^48^. To determine if the inhibition mechanism for SABA1 is the same as the mechanism of inhibition for triclosan, ADP was varied at fixed concentrations ATP and SABA1. The apparent inhibition constant (K_i_′) for the binding of SABA1 clearly decreases as the concentration of ADP increases (Figure 7) indicating uncompetitive inhibition with a K_i_ of 10.3 ± 0.1 µM. This result combined with the competitive pattern versus ATP reported above suggests that SABA1 inhibits BC by the same mechanism as triclosan inhibits EACPR.

### ACC inhibition patterns

**Figure 8.**
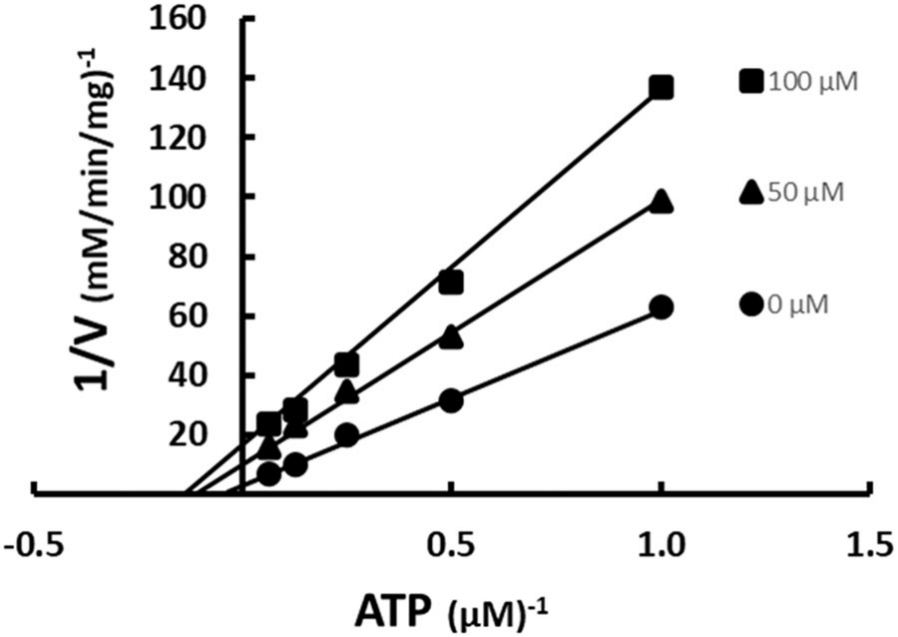
Inhibition of ACC by SABA1 with respect to ATP. The concentration of ATP was varied at fixed increasing concentrations of SABA1. Acetyl-CoA was held constant at subsaturating levels. Curves are the best fit of the data to Equation (2). Points are the experimentally obtained velocities.

ACC is the catalytically active structure *in vivo*^22^. As such, it is necessary to determine the inhibition patterns against that macromolecular complex. ATP was varied at fixed increasing concentrations of SABA1 while acetyl-CoA was held constant at subsaturating concentrations. SABA1 showed a non-competitive inhibition pattern with a K_is_ of 96.3 ± 31.6 µM and a K_ii_ of 23.3 ± 4.4 µM (Figure 8). These inhibition constants are lower than the ones obtained for the isolated BC subunit, indicating that SABA1 binds tighter to ACC than to isolated BC.

The above observations are consistent with a report from Silvers et al.^50^ which found that aminooxazole, a known BC inhibitor that binds in the ATP binding site^25^, is competitive versus ATP when assayed with the isolated BC subunit^50^. However, aminooxazole was non-competitive versus ATP when assayed with ACC^50^. Moreover, aminooxazole has a lower inhibition constant for ACC than for BC (0.4 ± 0.1 μM versus 0.8 ± 0.2 μM)^50^. A molecular explanation for both these results will have to wait for a 3 D structure of bacterial ACC.

## Discussion

### Biotin carboxylase inhibition

The results of this study definitively show that the antibacterial properties of SABA1 are due to inhibition of biotin carboxylase. Unlike all the known inhibitors of BC which bind in the ATP binding site^25–27^, SABA1 binds in the biotin-binding site. This is advantageous with respect to antibiotic development as humans have 49 different ATP-binding cassette transport proteins^51^, 95 helicases^52^, and over 500 kinases^53^ all of which bind and utilise ATP. The biotin carboxylase ATP binding site is structurally similar to the active sites in eukaryotic kinases^27^; therefore, any BC inhibitor that binds in the ATP binding site could potentially bind to one of the numerous eukaryotic ATP-utilising proteins. In fact, pyridopyrimidine which inhibits BC by binding in the ATP-binding site does significantly inhibit human scr kinase^27^.

### Mechanism of SABA1 inhibition

The mechanism by which SABA1 inhibits BC bears a striking similarity to the mechanism of inhibition of EACPR by triclosan. Triclosan exhibits competitive inhibition versus NADH, the first substrate to bind to EACPR^48^. SABA1 also exhibits competitive inhibition against the first substrate to bind to biotin carboxylase, ATP (Figure 3). Yet, neither triclosan nor SABA1 bind in the NADH or ATP binding sites of their respective enzymes. Triclosan binds in the enoyl substrate binding site of EACPR^49^, while SABA1 binds in the biotin-binding site of BC. NAD^+^, a product of the reaction catalysed by EACPR, strongly enhances triclosan binding, resulting in uncompetitive inhibition with respect to NAD^+ ^^48^. Likewise, ADP, a product of the reaction catalysed by BC, strongly enhances SABA1 binding, resulting in uncompetitive inhibition with respect to ADP. It is interesting to note that aminooxazole, a BC inhibitor that binds in the ATP binding site^25^, also enhances SABA1 binding.

The competitive inhibition of triclosan and SABA1 with respect to the enoyl substrate and biotin respectively, is explained by simple competition of the substrate and inhibitor for the same binding site. In contrast, the competitive inhibition of triclosan and SABA1 with respect to NADH and ATP is more nuanced. In the case of triclosan, NADH competes with NAD^+^ for binding to the free enzyme^47^, therefore at saturating concentrations of NADH, NAD^+^ cannot bind to free EACPR. If NAD^+^ is not bound to EACPR, the binding affinity of triclosan is markedly decreased^48^. In other words, when NADH is bound to EACPR, NAD^+^, and therefore, triclosan cannot bind. This makes the binding of NADH and triclosan mutually exclusive. This mutual exclusivity manifests as competitive inhibition of triclosan versus NADH. The explanation for the SABA1 data follows an analogous pattern (Scheme 2). ATP competes with ADP for binding to BC. At saturating ATP, ADP is not bound, thereby decreasing the binding affinity of SABA1 to BC. In other words, when ATP is bound to BC, ADP, and therefore, SABA1 cannot bind. This makes the binding of ATP and SABA1 mutually exclusive. Thus, the observed competitive inhibition of SABA1 versus ATP.

### Docking results

A 3 D-structure could provide a molecular explanation for why ATP prevents SABA1 binding while ADP enhances SABA1 binding. However, despite numerous attempts, a crystal structure of SABA1 bound to BC is not available. In order to gain insight into the possible molecular explanation of the observed kinetic results, SABA1 was docked into the biotin-binding site of either free BC, or BC bound to ADP and AMPPCP. While docking results are no substitute for structural data, they can provide a working hypothesis for future structure-function analyses.

**Figure 9.**
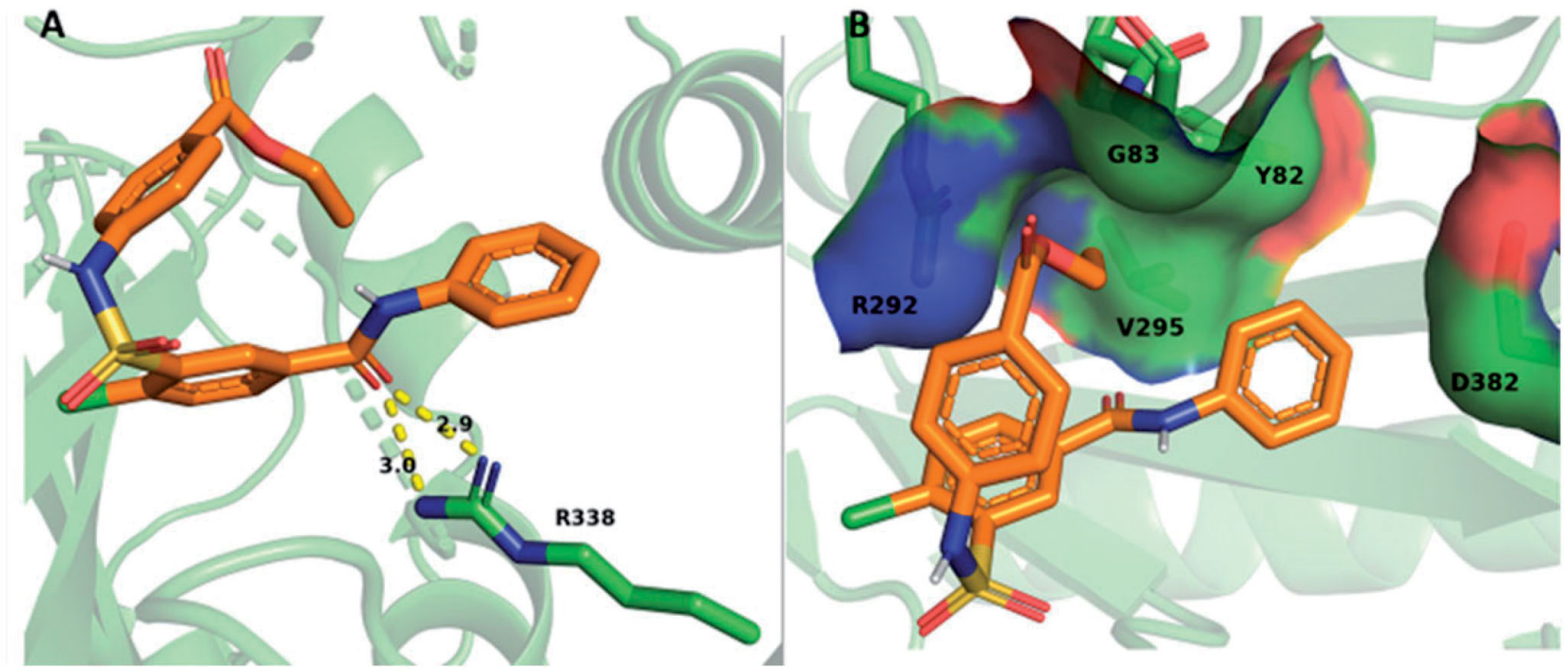
SABA1 docked into the biotin binding pocket of free BC. (A) R338 hydrogen bonds to the carbonyl oxygen in the benzylamide of SABA1. Yellow dashes represent hydrogen bonds, lengths are in Å. (B) Partial surface representation showing the interactions between the ethyl ester and phenyl group of SABA1 with the positive pocket and hydrophobic patch of BC respectively. In the surface representation, red is oxygen, blue is nitrogen, and green is carbon. A and B were generated with PyMOL.

SABA1 docked onto free BC, adopts a folded conformation (Figure 9(A)) with a predicted binding affinity of −6.3 kcal/mol. For reference, the carboxybiotin analogue, N1′-methoxycarbonyl biotin methyl ester, binds in the biotin binding site^38^ has a predicted binding affinity of −6.3 kcal/mol. R338 forms a hydrogen bond with the carbonyl oxygen of the benzylamide in SABA1 (Figure 9(A)). The ethyl ester of SABA1 sits in a positively charged pocket formed by the side chain of R292 and the main chain amide of G83 (Figure 9(B)). The phenyl ring of SABA1 is juxtaposed between V295, Y82, and the β-carbon of D382 (Figure 9(B)).

**Figure 10.**
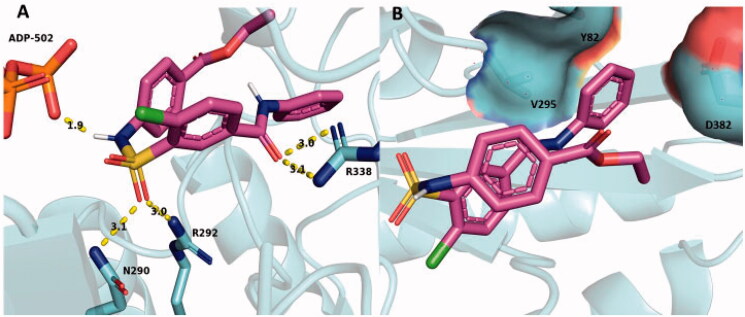
SABA1 docked into the biotin binding pocket of BC bound to ADP. (A) Hydrogen bonds from BC and ADP to SABA1. Yellow dashes represent hydrogen bonds, lengths are in Å. (B) Partial surface representation showing the interactions between the phenyl group of SABA1 and hydrophobic patch of BC. In the surface representation, red is oxygen, blue is nitrogen, and green is carbon. A and B were generated with PyMOL.

When SABA1 was docked onto BC with ADP bound, the predicted binding affinity increased to −6.8 kcal/mol. It is important to note that the predicated binding affinity of SABA1 in the ADP bound structure (−6.8 kcal/mol) is tighter than the predicted binding affinity of the carboxybiotin analogue, N1′-methoxycarbonyl biotin methyl ester, to BC (−6.3 kcal/mol). The N1′-methoxycarbonyl biotin methyl ester is known to bind in the biotin binding site^38^. This suggests that SABA1 binds stably to BC in the presence of ADP. R338 again forms a hydrogen bond to the carbonyl oxygen of the benzylamide in SABA1 (Figure 10(A)) and the phenyl ring is again juxtaposed between V295, Y82, and the β-carbon of D382 (Figure 10(B)). However, to accommodate the β-phosphate of ADP the sulphonamide group of SABA1 was shifted 3.2 Å away from its position in the ADP free structure. This shift positions the carbonyl oxygens of the sulphonamide group within hydrogen bonding distance of R292 and N290. In addition, the amide nitrogen of the sulphonamide group forms a hydrogen bond with of the β-phosphate of ADP (Figure 10(A)). These docking results could explain the uncompetitive inhibition data versus ADP. When ADP is not present, SABA1 forms few strong interactions with BC. However, when ADP is present the new position of the sulphonamide group allows it to form hydrogen bonds with R292 and N290, along with the β-phosphate of ADP increasing the affinity of SABA1 for BC.

**Figure 11.**
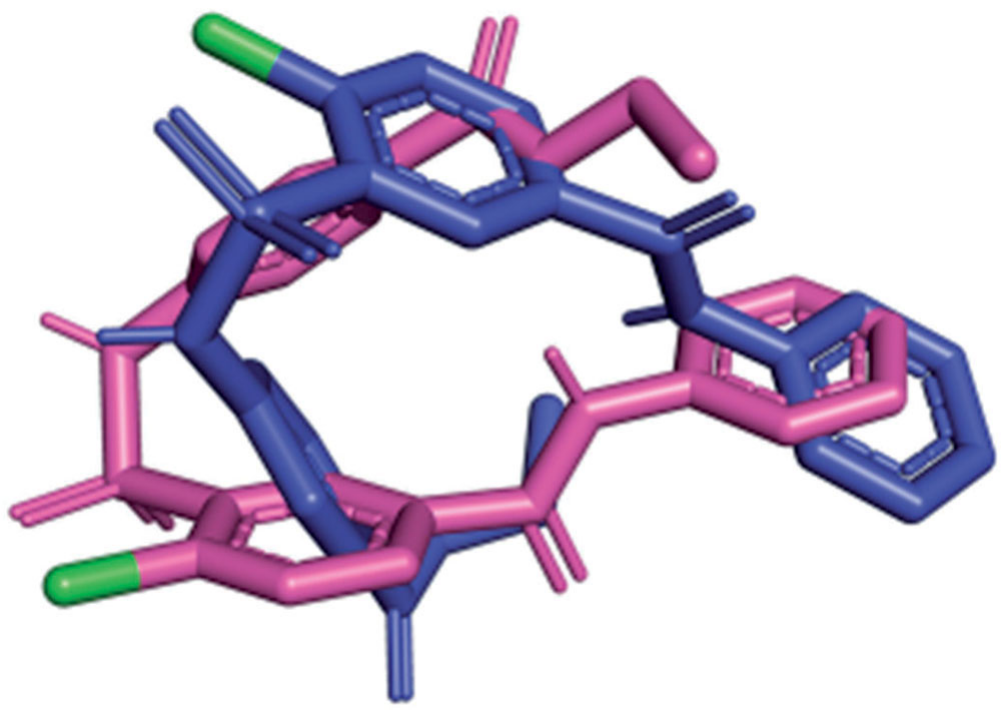
SABA1 in the presence of AMAPCP (blue) was overlayed onto SABA1 in the presence of ADP (magenta). Note the position of the chlorine atoms (green) is flipped. Generated with PyMOL.

The kinetic data show that SABA1 does not bind in the presence of ATP. In order to determine a possible molecular mechanism that explains why ATP prevents SABA1 binding, SABA1 was docked onto BC with ATP analogue AMPPCP bound. With AMPPCP bound SABA1 adopts an inverted conformation with respect to the conformation it had with ADP bound (Figure 11). This inversion results in the amide nitrogen of the benzylamide not being within hydrogen bond distance of R338. The γ-phosphate of AMPPCP caused the sulphonamide group of SABA1 to be positioned 5.6 Å away from its position in the ADP-bound structure (Figure 11). The position of the sulphonamide group is not within hydrogen-bonding distance of R292 or N290. In addition, none of the phosphate groups of AMPPCP are within hydrogen-bonding distance of the sulphonamide group of SABA1.

The docking results could provide an explanation of how SABA1 can simultaneously appear competitive versus ATP and uncompetitive versus ADP. ADP is necessary for efficient SABA1 binding as it positions the sulphonamide group in a favourable conformation to hydrogen bond to R292 and N290. In addition, the β-phosphate of ADP hydrogen bonds to SABA1 further enhancing the binding affinity. On the other hand, the presence of ATP forces SABA1 into an inverted conformation in which the sulphonamide group is not able to form hydrogen bonds to strengthen binding to BC.

The residues involved in hydrogen bonding between SABA1 and ADP-bound BC (N290, R292, and R338) are all involved in BC catalysis^29^^,^^38^^,^^54^. N290A and R292A mutations decrease the activity of BC 250-fold and 200-fold respectively^29^, while a R338Q mutation decreases BC activity 100-fold^54^. Thus, mutation of those residues to generate resistance to SABA1 binding would be lethal to the bacterium.

### Summary

This report definitively shows that SABA1 does inhibit BC and it does so with an atypical mechanism. Moreover, SABA1 is the first known inhibitor of BC with antibacterial properties that does not bind in the ATP-binding site. All other known BC inhibitors with antibacterial properties bind in the ATP-binding site. Since, biotin carboxylase is highly conserved amongst bacteria species^55^ and given that SABA1 is already effective against Gram-negative bacteria^28^, it could serve as a scaffold for the development of novel antibiotics against the Gram-negative ESKAPE pathogens.
